# Expression of Chrna9 is regulated by Tbx3 in undifferentiated pluripotent stem cells

**DOI:** 10.1038/s41598-023-28814-7

**Published:** 2023-01-28

**Authors:** Takashi Yazawa, Yoshitaka Imamichi, Takeshi Kitano, Mohammad Sayful Islam, Md. Rafiqul Islam Khan, Satoru Takahashi, Toshio Sekiguchi, Nobuo Suzuki, Akihiro Umezawa, Junsuke Uwada

**Affiliations:** 1 Department of Biochemistry, Asahikawa Medical University, Midorigaoka Higashi 2-1-1-1, Asahikawa, Hokkaido 078-8510 Japan; 2 Department of Marine Science and Technology, Faculty of Marine Science and Technology, Fukui Prefectural University, Fukui, 917-0003 Japan; 3 Department of Biological Sciences, Graduate School of Science and Technology, Kumamoto University, Kumamoto, 860-8555 Japan; 4 Department of Pharmacy, University of Rajshahi, Rajshahi, Bangladesh; 5 Department of Pediatrics, Asahikawa Medical University, Midorigaoka Higashi 2-1-1-1, Asahikawa, Hokkaido 078-8510 Japan; 6 Noto Marine Laboratory, Division of Marine Environmental Studies, Institute of Nature and Environmental Technology, Kanazawa University, Noto-cho, Ishikawa 927-0553 Japan; 7 Department of Reproduction, National Center for Child Health and Development Research Institute, Setagaya, Tokyo 157-8535 Japan; 8 Department of Pharmacology, School of Medicine, Kanazawa Medical University, Uchinada, Ishikawa Japan

**Keywords:** Developmental biology, Stem cells

## Abstract

It was reported that nicotinic acetylcholine receptor (nAChR)-mediated signaling pathways affect the proliferation and differentiation of pluripotent stem cells. However, detail expression profiles of nAChR genes were unrevealed in these cells. In this study, we comprehensively investigated the gene expression of α subunit of nAChRs (Chrna) during differentiation and induction of pluripotent stem cells. Mouse embryonic stem (ES) cells expressed multiple Chrna genes (Chrna3-5, 7 and 9) in undifferentiated status. Among them, Chrna9 was markedly down-regulated upon the differentiation into mesenchymal cell lineage. In mouse tissues and cells, Chrna9 was mainly expressed in testes, ES cells and embryonal F9 teratocarcinoma stem cells. Expression of Chrna9 gene was acutely reduced during differentiation of ES and F9 cells within 24 h. In contrast, Chrna9 expression was increased in induced pluripotent stem cells established from mouse embryonic fibroblast. It was shown by the reporter assays that T element-like sequence in the promoter region of Chrna9 gene is important for its activities in ES cells. Chrna9 was markedly reduced by siRNA-mediated knockdown of Tbx3, a pluripotency-related transcription factor of the T-box gene family. These results indicate that Chrna9 is a nAChR gene that are transcriptionally regulated by Tbx3 in undifferentiated pluripotent cells.

## Introduction

Pluripotent stem cells, such as embryonic stem (ES) cells, maintain their pluripotency and undifferentiated status through a molecular network of transcription factors. Analysis of this network has identified three central factors, such as Oct-3/4, Sox2, and Nanog^1^. These transcription factors are highly expressed in pluripotent cells, such as the inner cell mass, epiblast, and ES cells. Null mutations and repression of these genes induce early embryonic lethality and differentiation failure because of an inability to maintain pluripotency^2–5^. In addition to these central factors, other transcription factors play supporting roles. Among them, Klf4 and Tbx3 represent such factors that function via the interaction with each central factor. Artificial expression of Klf4 and Tbx3 is sufficient to maintain the undifferentiated status in murine ES cells without leukemia inhibitory factor (LIF), a cytokine that are essential for maintaining the self-renewal and pluripotency^6^. Along with Oct-3/4 and Sox2, Klf4 is essential for reprograming the terminal differentiated somatic cells to induced pluripotent stem (iPS) cells^7^. Although Tbx3 never affects the reprograming efficiency in establishment of iPS cells, it can improve the germ-cell contribution to the gonads and germ-line transmission frequency of iPS cells generated by Oct-3/4, Sox2 and Klf4^8^.

Nicotinic acetylcholine receptors (nAChRs) belong to the Cys-loop family of pentameric ligand-gated ion channels. They are composed of various α (Chrna1-10, but Chrna8 is an avian-specific) and β (Chrnb1–4) subunits^9,10^. Although they can constitute various heteromeric pentamers, α subunits (Chrna) are essential for responding to the ligands. Some α subunits (Chrna7 and Chrna9) can function as homomeric pentamers^11,12^. Upon ligand-binding, pentameric receptors undergo conformational changes to open a central pore, causing the influx of extracellular ions and following various cellular responses.

nAChR-mediated signaling pathways play important roles in neuronal tissues^13,14^. Knockout (KO) mice of multiple Chrna genes exhibited the phenotypes of abnormalities in nervous systems^15–26^. In addition to neuronal tissues, Chrna and Chrnb genes are expressed in various non-neuronal tissues and cell types^27,28^. It was reported in previous studies that various Chrna and Chrnb genes are expressed in pluripotent stem cells, such as embryonic stem (ES) cells^29,30^ and induced pluripotent stem (iPS) cells^31,32^. ACh and nicotine affect the proliferation and survival of these pluripotent stem cells via these receptors. Nicotine increases the DNA synthesis via Chrna4 and Chrna7 in iPS cells^31,32^. In ES cells, high dose-ACh and -nicotine reduce the apoptosis, whereas they inhibit the proliferation^29^. In contrast, it is unknown whether nAChR-mediated signaling pathways are involved in undifferentiation status and pluripotency of pluripotent stem cells. In this study, we investigated the expression of Chrna genes upon the differentiation and establishment of pluripotent stem cells. We demonstrated that Chrna9 is expressed in undifferentiated pluripotent stem cells.

## Results

### Expression of Chrna genes during differentiation of ES and EC cells

**Figure 1:**
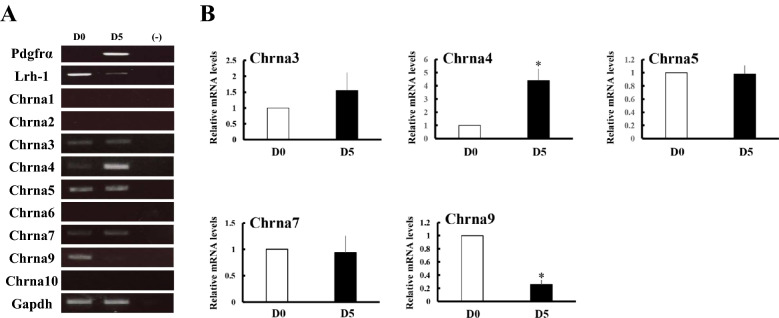
Expression of Chrna genes during differentiation of ES cells into mesenchymal cell lineage. ES cells were cultured on collagen IV-coated dishes with differentiation media for 5 days. (**A**) RT-PCR analyses of each gene in ES cells (D0) and differentiated cells (D5). (**B**) Expression of each Chrna gene was analyzed in ES cells (D0) and differentiated cells (D5) by qPCR and normalized to 36B4 expression. Data represent the mean ± SEM of four independent experiments. Differences between groups are indicated by *P < 0.05.

We investigated the expression of Chrna genes in murine ES cells by RT-PCR (Fig. 1A). Among nine Chrna genes, ES cells expressed Chrna3, Chrna4, Chrna5, Chrna7 and Chrna9. To investigate the fluctuation of these genes upon differentiation, ES cells (D0) were differentiated into mesenchymal cell lineage including MSCs by culturing on collagen type IV-coated dishes and treating with pulse exposures of retinoic acid (RA) for 5 days (D5). These cells expressed platelet-derived growth factor receptor α (Pdgfrα), one of the molecular markers for the mesenchymal cell lineage^33^, whereas expression of liver receptor homolog-1 (Lrh-1), one of the undifferentiated marker genes in ES cells^34^, was declined (Fig. 1A). Upon differentiation, expression of Chrna4 mRNA was increased, whereas Chrna9 expression was markedly decreased (Fig. 1A,B). Expression levels of Chrna3, Chrna5 and Chrna7 were almost constant. Expression of other Chrna genes was very low or undetectable levels regardless of differentiation status.

**Figure 2:**
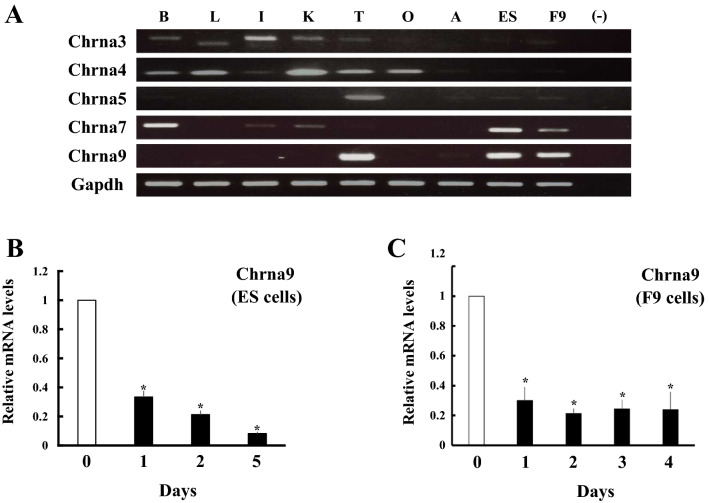
Expression of Chrna9 in murine tissues and pluripotent stem cells. (**A**) Expression of Chrna genes in murine tissues and pluripotent stem cells. mRNA expression of each gene was analyzed in brain (lane B), liver (lane L), intestine (lane I), kidney (lane K), testis (lane T), ovary (lane O), adrenal (lane A), ES cells (lane ESC) and F9 cells (lane F9) by RT-PCR. (**B**) Expression of Chrna9 during differentiation of ES cells into mesenchymal stem cells. Expression of Chrna9 gene was analyzed by qPCR and normalized to 36B4 expression. Data represent the mean ± SEM of four independent experiments. *P < 0.05 vs. day 0. (**C**) Expression of Chrna9 during differentiation of F9 cells into parietal endodermal cells. Expression of Chrna9 gene was analyzed by qPCR and normalized to 36B4 expression. Data represent the mean ± SEM of four independent experiments. *P < 0.05 vs. day 0.

Next, we investigated the expression of these genes in various tissues and stem cells of mice (Fig. 2A). Chrna3 and Chrna4 were expressed in various tissues. Chrna5 is strongly expressed in testis. These genes were also detectable in ES cells and embryonal carcinoma/teratocarcinoma stem cell F9 cells, albeit at lower levels. Although Chrna7 was strongly expressed in brain, ES cells and F9 cells, it was also detectable at lesser extent in other tissues, such as colon, kidney and testis. Chrna9 was strongly expressed in testis, ES cells and F9 cells. These results indicate the possibility that Chrna9 represents one of the marker genes in undifferentiated pluripotent stem cells.

In support of this hypothesis, Chrna9 expression was acutely down-regulated in ES cells and F9 cells within 24 h upon induction of mesenchymal cell lineage and parietal endodermal cells, respectively (Fig. 2B,C). It was maintained at low levels during differentiation.

### Expression of Chrna genes in MEF-derived iPS cells

**Figure 3:**
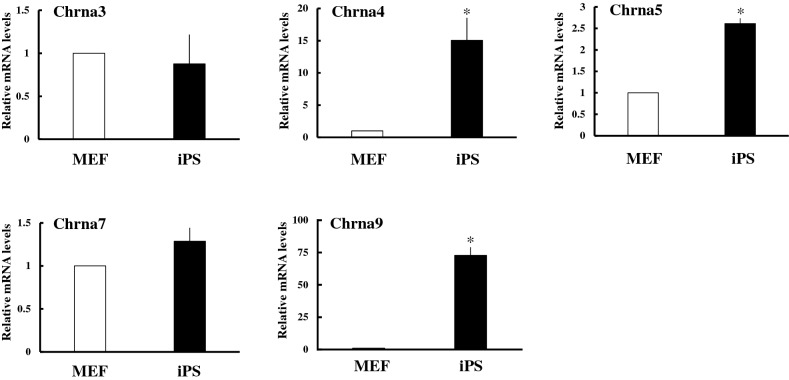
Comparison of *Chrna* gene expression between MEF and MEF-derived iPS cells. Expression of each *Chrna* gene was analyzed in MEF and iPS cells by qPCR and normalized to 36B4 expression. Data represent the mean ± SEM of four independent experiments. Differences between groups are indicated by *P < 0.05.

To further confirm above hypothesis, we investigated whether Chrna9 is induced upon reprograming of terminal differentiated cells using MEF-derived iPS cells established by introducing Yamanaka factors^35^ (Fig. 3). Although Chrna9 mRNA levels were very low in MEF, it was strongly expressed in iPS cells at over 70-fold. Expression of Chrna4 and Chrna5 mRNA were also higher in iPS cells than in MEF, albeit to a lesser extent. Expression of Chrna3 and Chrna7 was similar levels in MEF and iPS cells.

### Transcriptional regulation of Chrna9 in ES cells

**Figure 4:**
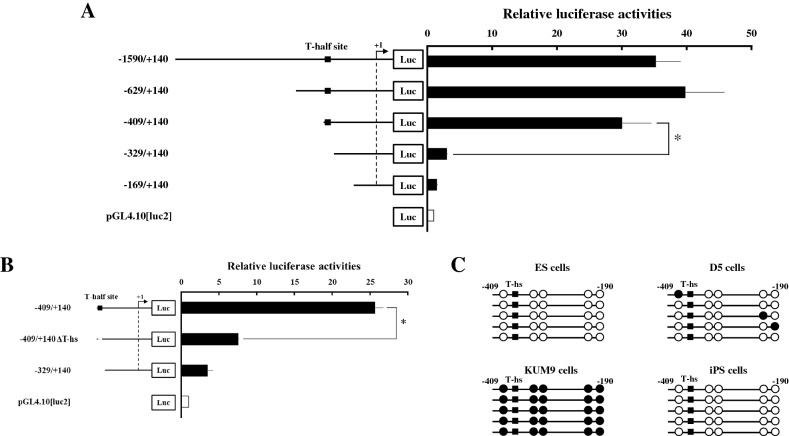
Analyses of mouse *Chrna9* promoter in ES cells. (**A**) 5′-deletion analysis of the mouse Chrna9 promoter region. Progressive deletions of the upstream region are illustrated schematically in the left panel. Each vector was transfected by lipofection into ES cells. At 24 h after transfection, luciferase assays were performed using the cell lysates. Relative luciferase activities are shown. Data are the mean ± SEM values of at least four independent experiments; and *, *P* < 0.05. (**B**) Effects of deletion in the T-half site within the Chrna9 promoter region. The constructs used are drawn schematically. Transfection and luciferase assays were performed as described in A. Data are the mean ± SEM values of at least four independent experiments; and *, *P* < 0.05. (**C**) Methylation analysis of the promoter region (− 409 to − 190) of *Chrna9* gene in undifferentiated ES, differentiated (D5), KUM9 and iPS cells. Each circle denotes cytosine bases in CpG dinucleotides, and *filled* and *open circles* represent methylated and unmethylated cytosines, respectively.

To study transcriptional regulation of Chrna9 gene in pluripotent stem cells, a reporter plasmid containing the 5′-flanking region (− 1590 bp) of the mouse Chrna9 gene was transfected into ES cells (Fig. 4). Promoter activities of the Chrna9 were over 30-fold higher compared with transfection of the pGL4.10[luc2] empty vector in ES cells (Fig. 4A). In contrast, it was not activated in mouse mesenchymal stem cell-derived KUM9 cells that were not express Chrna9 mRNA (Supplementary Fig. 1). To define the cis-regulatory element(s) required for the promoter activities, deletion analyses were performed. Promoter activity was retained at the − 409 fragment, although it was nearly abolished by the truncation to − 329. Examination of this sequence using JASPAR (https://jaspar.genereg.net/) revealed the presence of T-half site-like sequence (AGGTGTaAA) at the position − 396/ − 388 (Fig. 4B). Deletion of this sequence substantially decreased promoter activity, similar to that for the − 329 fragment. These findings indicate that T-half site sequence is essential for the transcription of Chrna9 gene in ES cells. Consistent with this, cytosine residues surrounding this region was completely unmethylated in ES cells (Fig. 4C). However, it was partially methylated after upon differentiation. This region was completely methylated in KUM9 cells, whereas it was completely unmethylated in iPS cells.

**Figure 5:**
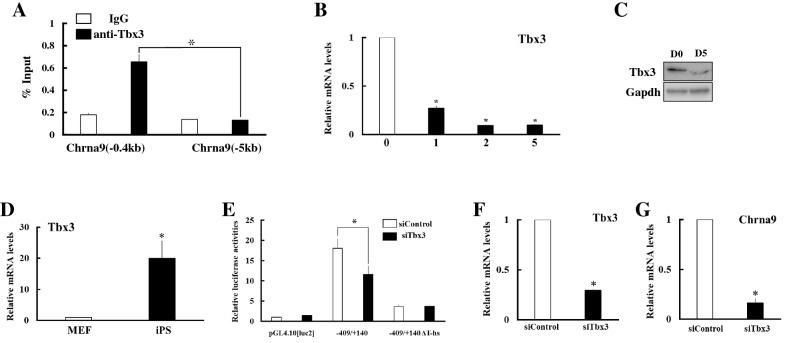
Involvement of Tbx3 in the transcriptional regulation of *Chrna9* in pluripotent stem cells. (**A**) ChIP assays showing that Tbx3 occupied its binding site on the promoter region of *Chrna9*. Assays were performed on ES cell extracts using control IgG or anti-TBX3 antibody. Recovered chromatin was subjected to qPCR analysis using primers encompassing proximal Tbx3 binding site (− 0.4 kb) or 5 kb from the transcription start site (− 5 kb). ^⁎^*P* < 0.05 between the indicated groups. (**B**) Expression of Tbx3 during differentiation of ES cells into mesenchymal cells. Expression of Chrna9 gene was analyzed by qPCR and normalized to 36B4 expression. Data represent the mean ± SEM of four independent experiments. *P < 0.05 vs. day 0. (**C**) Western blot analyses were conducted in ES cells before (D0) and after differentiation (D5), using antibodies against Tbx3 and Gapdh. (**D**) Expression of Tbx3 was analyzed in MEF and iPS cells by qPCR and normalized to 36B4 expression. Data represent the mean ± SEM of four independent experiments. Differences between groups are indicated by *P < 0.05. (**E**) Effects of siRNA-mediated Tbx3 knockdown on the promoter activities of Chrna9 gene. Each reporter construct was co-transfected to ES cells with control siRNA (white boxes) or Tbx3 siRNA (black boxes). To investigate the effects of Tbx3 knockdown, luciferase assays were performed using the cell lysates at 48 h after transfection. Relative luciferase activities are shown. Data are the mean ± SEM values of at least four independent experiments; and *, *P* < 0.05. (**F**, **G**) Effects of siRNA-mediated Tbx3 knockdown on the expression of Tbx3 (**F**) and Chrna9 (**G**) in ES cells. Expression of each gene was analyzed by qPCR and normalized to 36B4 expression. Data represent the mean ± SEM of four independent experiments. Differences between groups are indicated by *P < 0.05.

T-elements and its half sites are occupied by the TBX transcription factors that include at least 17 family members. Among these family members, Tbx3 plays important roles to maintain and induce pluripotency of ES cells and iPS cells by regulating the transcription of various pluripotent-associated genes. ChIP assays showed that Tbx3 binds to promoter region of Chrna9 gene in ES cells, whereas the specific-binding was not detectable − 5 kb upstream of the transcription start site (Fig. 5A). In addition, its expression was acutely down-regulated to about one-fourth in ES cells within 24 h upon induction of mesenchymal cell lineage and continued thereafter (Fig. 5B). Tbx3 protein was barely detectable after differentiation (Fig. 5C). In contrast, it was strongly expressed in iPS cells than in MEF at about 30-fold (Fig. 5D).

To investigate the contribution of Tbx3 to Chrna9 expression in pluripotent stem cells, the knockdown experiments were performed using Tbx3 siRNA in ES cells. Luciferase reporter assays showed that co-transfection of Tbx3 siRNA significantly reduced Chrna9 promoter activity in the − 409 fragment, whereas it unaffected the activity of the T-half site deleted fragment (Fig. 5E). In addition, siRNA mediated-knockdown of Tbx3 markedly decreased Chrna9 expression to 16% (Fig. 5F,G). In contrast, expression of other Chrna genes was not significantly affected by Tbx3 knockdown (Supplementary Fig. 2). In addition, transfection of Tbx3 expression vector increased Chrna9 expression, even though it was not completely rescued the down-regulation by the induction of differentiation (Supplementary Fig. 3). These results strongly suggest that Tbx3 is a main regulator of Chrna9 expression in pluripotent stem cells.

## Discussion

We have comprehensively analyzed the expression of Chrna genes in murine ES cells, and revealed that five Chrna genes (Chrna3, Chrna4, Chrna5, Chrna7 and Chrna9) were expressed. Among them, Chrna9 expression was maintained in undifferentiated pluripotent stem cells and quickly down-regulated upon differentiation. This expression pattern is likely caused by the transcriptional regulation via Tbx3. Chrna9 is known to be expressed in relatively limited tissues and cells, such as cochlear outer hair cells^36^, dorsal root ganglia^37^, keratinocytes^38^, lymphocytes^39^ and testis^40^. However, there are few reports focusing on its expression in early embryonic development. Our study provides novels insights into expression and possible functions of Chrna9 gene.

Chrna9 can assemble into a functional homopentamers that are coupled to calcium‐activated potassium channels^11,12^. It can also assemble to Chrna10 to form heteropentamers^41^. However, because Chrna10 was undetectable in murine pluripotent stem cells, Chrna9 likely functions as homopentamers. Upon ligand-binding to Chrna9-containg pentamers, a large Ca^2+^ influx occurs. It is well-known that the mobilization of intracellular Ca^2+^ play vital roles in proliferation, survival and differentiation of pluripotent stem cells^42^. Chrna9 might be involved in these phenomena, especially in pluripotency and undifferentiated status, by regulating intracellular Ca^2+^ concentrations. In addition to Ca^2+^-mediated signaling, it is possible that PI3K/AKT pathway is important for the functions of Chrna9 in ES cells. It was reported in melanoma cells that stimulation of Chrna9 with nicotine activates AKT^43^. Because activation of AKT signaling is sufficient for maintaining the pluripotent status in ES cells^44^, it is noteworthy to investigate the roles of Chrna9 and this pathway in pluripotent stem cells in the future study. However, Chrna9 is not essential at least in early development, because its KO mice develop to adult stage^45^. It is likely compensated by other factors, including various Chrna genes that are expressed in ES cells. This hypothesis might be supported by the facts that all available nAChR KO mice are survive during embryonic stage. However, each nAChR KO mice exhibit various phenotypes from postnatal days that are mainly caused by the problems of nervous systems^46^. Chrna9 KO mice shows abnormal cochlear efferent innervation, resulting in the suppression of cochlear responses^47^.

In contrast to Chrna9, Chrna4 expression was slightly increased during differentiation of ES cells. This result is inconsistent with a previous report by Kaltwasseer and colleague that Chrna4 was down-regulated upon LIF removal from culture media for 5 days in another murine ES cell line, CGR8^30^. Hence, they hypothesized that Chrna4 is an important nAChR in the regulation of pluripotency in murine ES cells. However, we showed that expression of Chrna4 was much higher in various tissues than in ES cells. In addition, Ishizuka et al. demonstrated that Chrna4 expression was constant in iPS cells cultured without LIF for 24 h^32^. These facts indicate that down-regulation of Chrna4 expression by LIF removal for 5 days is attributed to the differentiation of ES cells into some specific cell lineage, which has lower Chrna4 expression. Because Chrna4 expression was higher in iPS cells than in MEF, it can not be ruled out the possibility that it is involved in the pluripotency and undifferentiation status. However, tissue and cell non-specific expression pattern indicates that it has another role in pluripotent stem cells. In support of this hypothesis, it was reported that Chrna4 signaling stimulated DNA synthesis in iPS cells^31,32^. However, as in the case of other Chrnas, Chrna4 is not essential for early development. *Chrna4* KO mice survive until adult stage, despite they show the behavior with increased anxiety^48^.

In mouse pluripotent stem cell, Tbx3 is likely a main transcription factor to regulate expression of Chrna9. Pluripotent stem cells maintain their pluripotency and undifferentiated status through a molecular network of transcription factors^1^. Among them, Oct-3/4, member of the POU transcription factor family, is central factor for governing pluripotency. The fate of ES cells is determined by its expression level. Therefore, Oct-3/4 regulates its own expression in cooperation with Sox2, Nanog and other members of the transcriptional regulatory circuitry. Tbx3 represents such members and contributes to maintain the undifferentiated status of mouse ES cells by keeping Oct-3/4 expression via induction of Nanog^6^. Its knockdown causes downregulation of pluripotent-associated genes, leading to a loss of self-renewal and differentiation^8,49^. Tbx3 can also improve quality of iPS cells and increase frequency of germ line transmission^8^. Chrna9 may contribute to these processes as one of the target genes for Tbx3 in pluripotent stem cells. It is interesting that Tbx3 expression is maintained by PI3K/AKT pathway in ES cells^50^. Because this pathway is possible to activate by ligand-stimulated Chrna9^43^, Tbx3 and Chrna9 may positively control the expression of each other for contributing to maintain the pluripotency in ES cells. In contrast to early embryonic stage and ES cells, expression pattern of Tbx3 is inconsistent with Chrna9 in the developing and adult tissues. Tbx3 is expressed in various tissues, such as liver^51^, hearts^52,53^ and mammary glands^54,55^ that Chrna9 expression is undetectable. This fact strongly suggests that expression of Tbx3 is not involved in the Chrna9 expression in adult stage, as in the case of the pluripotent-associated genes in ES cells. It is remarkable that Tbx3 have both abilities to activate and repress gene transcription via binding to T-elements or its half site^56,57^. Therefore, it is possible that Tbx3 represses Chrna9 expression in adult tissues. Another possibility is that methylation of promoter region can contribute to this phenomenon, as was observed in differentiated ES cells and mesenchymal stem cell-derived KUM9 cells. In addition, it was reported in human CHRNA9 gene that activation and repression elements in the 5′-nonconding region play important roles for transcriptional regulation in CHRNA9-expressing human neuroblastoma SH-SY5Y cells^58^. Although it is interesting that there are the potential SOX2-binding sites in activation elements of human CHRNA9 gene, these sites are not conserved in rodents. Hence, transcription factors and transcription elements for regulating transcription of CHRNA9/Chrna9 gene may be completely different between early embryonic stage and later stage. It is necessary in future study to investigate transcriptional regulation of this gene in adult tissues and differentiated cells.

In summary, we demonstrated that Chrna9 is expressed in undifferentiated pluripotent stem cells via the transcriptional regulation by Tbx3. It is conceivable that Chrna9 could be one of the marker genes in ES cells and iPS cells. Our findings are important to reveal the roles of nAChR-mediated signaling pathways in pluripotent stem cells. In addition, these insights can provide some clues to understand the molecular mechanisms of pluripotency and fate determination in ES cells and iPS cells.

## Materials and methods

### Cell culture, transfection and luciferase assays

Culture methods for the murine ES cells (EBRTcH3) and the induction of mesenchymal stem cells (MSCs) have been described previously^59^. Briefly, they were cultured in Glasgow minimal essential medium (Sigma-Aldrich; St. Louis, MO, USA) supplemented with 10% fetal calf serum, 1 mM sodium pyruvate (Life Technologies Inc., Carlsbad, CA, USA), 100 µM 2-mercaptoethanol (Nacalai Tesque, Kyoto, Japan), 1 × nonessential amino acids (Life Technologies Inc.) and 1000 U/ml leukemia inhibitory factor (LIF; Wako Pure Chemical Industries Ltd., Osaka, Japan) on gelatin-coated dishes. Culture methods for the induction of MSCs have been described elsewhere^33,59^. ES cells were cultured on collagen IV-coated dishes without LIF for 2 days, followed by treating with retinoic acid (RA, 100 nM) for 3 days. Embryonal carcinoma (EC)/teratocarcinoma stem cell F9 cells, mouse embryonic fibroblast (MEF) and mouse mesenchymal stem cell line KUM9 cells were cultured in DMEM supplemented with 10% fetal bovine serum (FBS). F9 cells were induced to differentiate into the parietal endoderm by treating with RA (100 nM) and 8br-cAMP (1 mM) for 4 days. Murine iPS cells (iPS-MEF-Ng-20D-17, obtained from Riken BRC Cell Bank, Ibaraki, Japan)^35^ were cultured in iSTEM (Takara Bio Inc., Shiga, Japan). ES cells and KUM9 cells were transfected using Lipofectamine 2000 (Thermo Fisher Scientific Inc., MA, USA) or Hily MAX (Dojindo Laboratories, Kumamoto, Japan). At 24 h or 48 h post-transfection, luciferase assays were performed as described^60^. Measurements were made using a MiniLumat LB9506 (Berthold Systems, Aliquippa, PA, USA) in a single tube, with the first assay involving the firefly luciferase, followed by the *Renilla* luciferase assay. Firefly luciferase activities (relative light units) were normalized by *Renilla* luciferase activities. Each data point represents the mean of at least four independent experiments.

### RT-PCR and quantitative (q)PCR

Total RNA from the cultured cells and tissues was extracted using Tripure reagent (Roche Molecular Biochemicals, Mannheim, Germany). RT-PCR and qPCR were performed as described^61–63^. The RT-PCR products were subjected to electrophoresis on 1.5% (w/v) agarose gels, and the resulting bands were visualized by staining with ethidium bromide. In qPCR, each gene was measured via real-time PCR using the LightCycler 480 (Roche Diagnostics, Mannheim, Germany). 36B4 was used as an internal reference gene. Each reaction was conducted in duplicate. As a negative control, template cDNA was replaced by PCR grade water. Relative gene expression levels were determined by using the delta-delta Ct method. The primers used were in Supplementary Table 1. Other primers have been described previously^59,64,65^.

### Plasmids

A fragment containing 5′-region of murine Chrna9 gene (− 1590 to + 140) were amplified by genomic PCR and cloned into a pGL4.10[Luc2] vector (Promega, Carlsbad, CA, USA). The deletion constructs of Chrna9 promoter were amplified from the − 1590/ + 140 construct, sequenced, and cloned in the same vector. The pcDNA3 expressing mouse Tbx3 was generated by cloning the open reading frame of each gene into a pcDNA3 vector (Thermo Fisher Scientific).

### Methylation analyses

Bisulfite sequencing was performed for KUM9 cells-, EBRTcH3 cells- and iPS cells-derived genomic DNA samples treated with the Epitect Fast Bisulfite Kit (Qiagen, Hilden, Germany) that converts all the cytosines except for methylated cytosines at the CpG dinucleotides into uracils and subsequently thymines. Promoter region of Chrna9 gene surrounding T-half site like sequence was amplified by Takara Ex Premier DNA polymerase (Takara Bio Inc.) using the specific primers (Supplementary Table 1). After adding deoxyadenosines to 3´-each end, amplicons were subcloned with the pGEM-T easy vector (Promega), and multiple clones were subjected to direct sequencing on the ABI3500 Genetic Analyzer (Applied Biosystems, Waltham, MA, USA).

### ChIP assays

ChIP assays were performed as described^58^. Briefly, ES cells were cross-linked with 1% formaldehyde, rinsed with PBS and resuspended in SDS lysis buffer. Cell lysates were sonicated and immunoprecipitated with normal IgG or an anti-TBX3 antibody (A-6, Santa Cruz Biotechnology, CA, USA) using Protein G Mag Sepharose (Cytiva, Tokyo, Japan). Immunoprecipitated complexes were eluted with elution buffer. Then, the cross-links were reversed and DNA fragments were purified for analyzing by qPCR using the primers described in Supplementary Table 1.

### Western blot analysis

The extraction of protein and subsequent quantification was performed as described^63^. Equal amounts of protein (30 µg) were resolved by 10% SDS-PAGE and transferred to polyvinylidene difluoride membranes. Western blot analyses of Tbx3 and Gapdh were carried out with antibodies directed against TBX3 (A-6/A-20, Santa Cruz Biotechnology) and GAPDH (14C10; Cell Signaling Technology, Inc.). Clarity Western ECL Substrate (Bio-Rad Laboratories Inc., Hercules, CA, USA) were used for detection.

### Knockdown of Tbx3 by small interference RNA (siRNA)

siRNA experiments were performed as described previously^66^. Negative Control siRNA (AM4611; Thermo Fisher Scientific Inc.) or Tbx3 siRNA (s7477; Thermo Fisher Scientific Inc.) was transfected into ES cells with Lipofectamine RNAiMAX (Thermo Fisher Scientific Inc.) or Lipofectamine 2000 according to the manufacturer's instructions. The final siRNA concentration in the medium was 50 nM.

### Statistical analysis

Data are presented as the mean ± SEM. Differences between groups (*P* < 0.05) were assessed by the Student’s *t*-test or one-way ANOVA followed by Tukey’s multiple comparison test using EZR (Saitama Medical Center, Jichi Medical University, Saitama, Japan) which is a graphical user interface for R (The R Foundation for Statistical Computing, Vienna, Austria) as described^67,68^.

## Supplementary Information

Supplementary Information.

## Data Availability

All data generated or analysed during this study are included in this published article and its supplementary information files.
