# Supplementary material for: Expression of Chrna9 is regulated by Tbx3 in undifferentiated pluripotent stem cells
Source: Sci Rep. 2023 Jan 28;13:1611. doi: 10.1038/s41598-023-28814-7 (PMC9884305; doi:10.1038/s41598-023-28814-7)
Supplement: Supplementary file 1 — Supplementary Information. [file 41598_2023_28814_MOESM1_ESM.pdf]

Supplementary Table S1. Primers used in each experiment.

PCR primers	Forward primers	Reverse primers
**RT-PCR**		
mouse Chrna1	F-tgggctccgaacatgagacg	R-tggacgcaatgacaaagacc
mouse Chrna2	F-ctaccccgacgtcacctactac	R-ctctgtctcctccctttcttca
mouse Chrna3	F-gactaccaaaactgcaccatga	R-gggagaaggttcaaaaacacag
mouse Chrna4	F-gacttatcgaatccatgcacaa	R-atggagaggtctggtctgacac
mouse Chrna5	F-aaacgaaggtgaaaagattagc	R-catagaaacatccgatcgagaa
mouse Chrna6	F-gatgatgaggaaacctctggac	R-accaccatagccatgtatttcc
mouse Chrna7	F-ggccaacgactcgcagccgctc	R-gcaggtccaaggaccaccctc
mouse Chrna9	F-tctctgggagtgaccatcctat	R-ctggaaaggtctttgtttctgg
mouse Chrna10	F-ccctaatgcacatccagtacct	R-gaaaaagcggtccattactctg
**Q-PCR**		
mouse Chrna3	F-ccagatcatggaaaccaacc	R-catttcagcttgtagtcattcca
mouse Chrna4	F-tccgcatcccatctgaactc	R-agaacaggtgggctttggtt
mouse Chrna5	F-cagctaatgaccaccaacgtc	R-gagagtctgaaggaacacgtataattt
mouse Chrna7	F-gcagatcatggatgtggatg	R-caagacgttggtgtggaatg
mouse Chrna9	F-gtacgatgggctcatcacct	R-aaccaaaggtcaggttgcac
**Bisulfite**		
Chrna9	F-aaagtttggttgatttggta	R-ctatttaatttaaaaactac
**ChIP assay**		
-0.4kb	F-aaagcctggttgacttggta	R-gtactagtgactgatttaaa
-5kb	F-gtaagtaatgtaggtaaacc	R-ttcagaggttcagtccatta

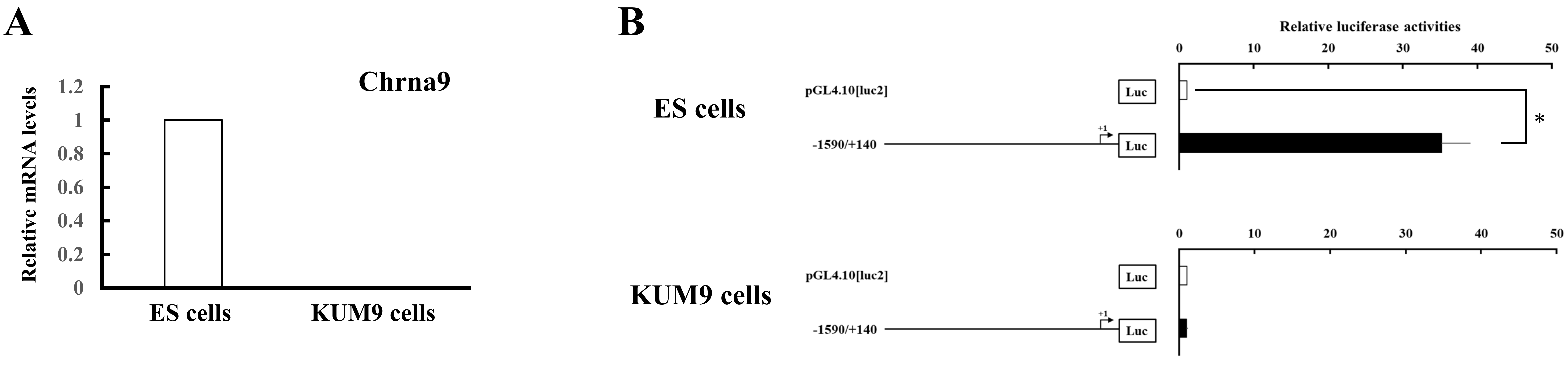

Supplementary Fig. 1. (A) Expression of each Chrna9 gene was analyzed in ES cells and KUM9 cells by qPCR and normalized to 36B4 expression. Data represent the mean ± SEM of four independent experiments. (B) Analyses of mouse Chrna9 promoter in ES cells and KUM9 cells. Each vector was transfected by lipofection. At 24 h or 48 h after transfection, luciferase assays were performed using the cell lysates. Relative luciferase activities are shown. Data are the mean ± SEM values of at least four independent experiments. Differences between groups are indicated by *$P < 0.05$.

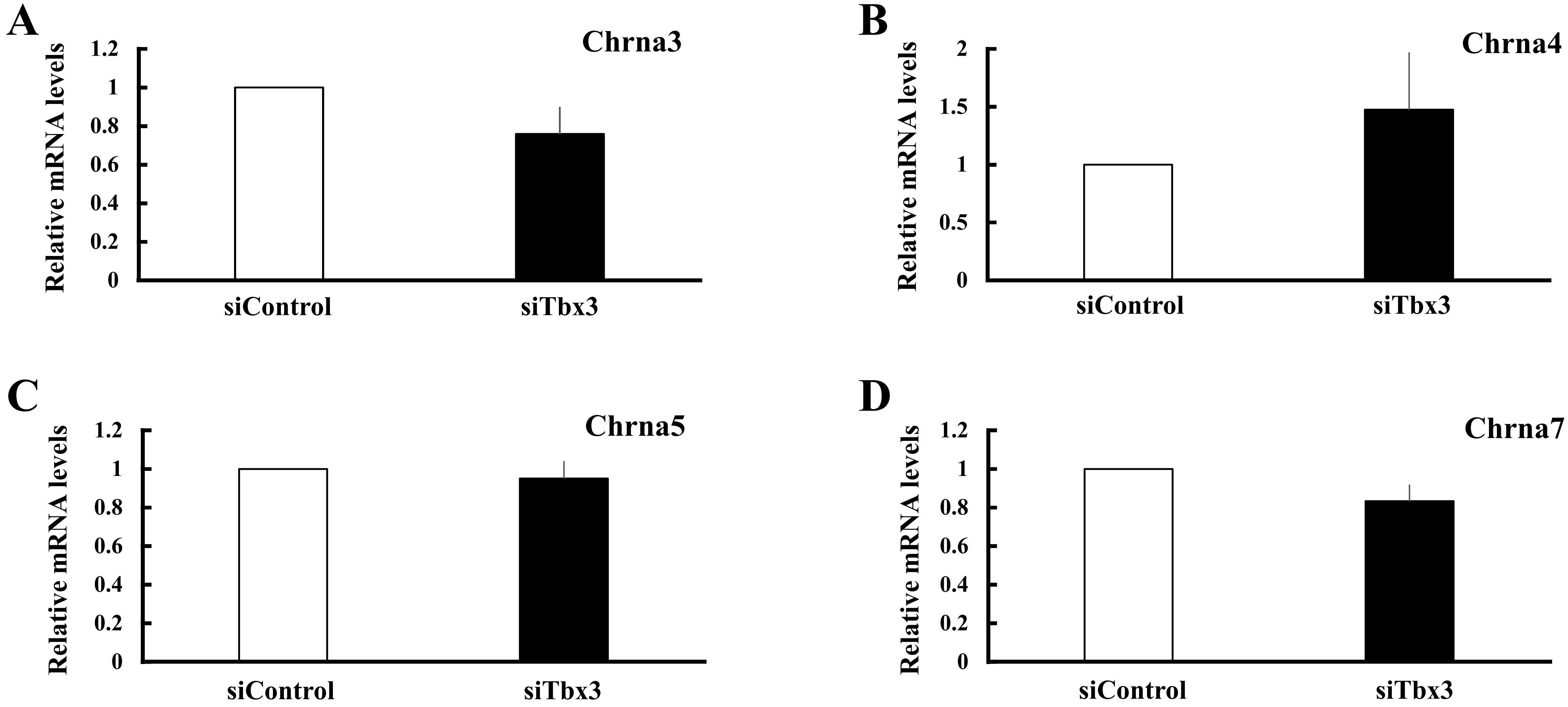

Supplementary Fig. 2. Effects of siRNA-mediated Tbx3 knockdown on the expression of Chrna3 (A), Chrna4 (B), Chrna5 (C) and Chrna7 in ES cells. Expression of each gene was analyzed by qPCR and normalized to 36B4 expression. Data represent the mean ± SEM of four independent experiments. There are no significant dfferences between groups.

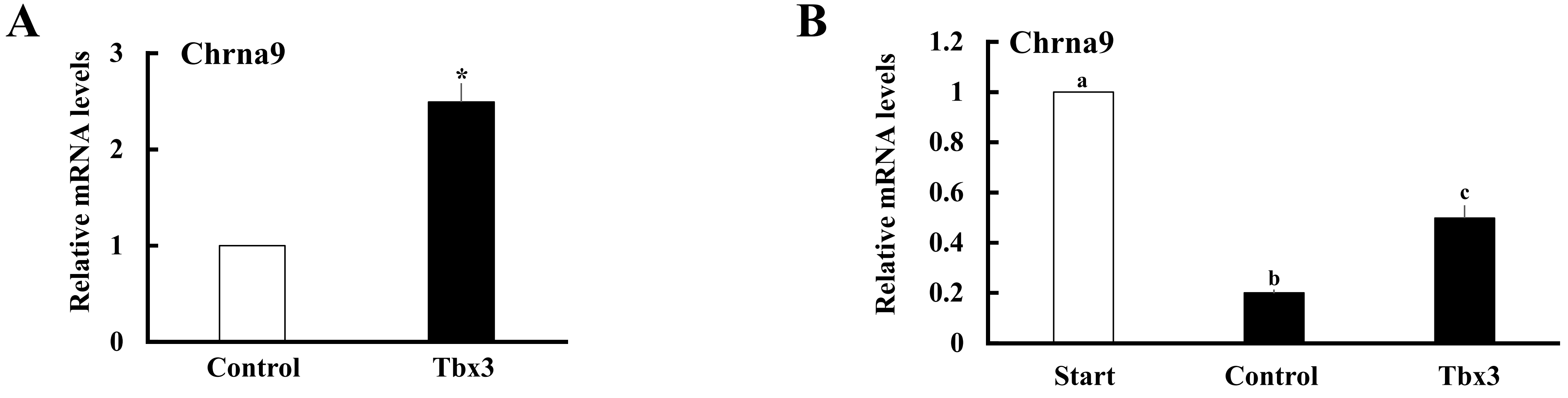

Supplementary Fig. 3. Effects of Tbx3 on the expression of Chrna9 during differentiation of ES cells. ES cells (Start) were transfected with pcDNA3 (control) or pcDNA3-Tbx3 (Tbx3), and cultured in differentiation media for 48 h. Expression of Chrna9 gene was analyzed by qPCR and normalized to 36B4 expression. Data represent the mean ± SEM of four independent experiments. (A) Differences between groups are indicated by *$P < 0.05$. (B) Values marked by the different letters are significantly different from each other ($P < 0.05$).

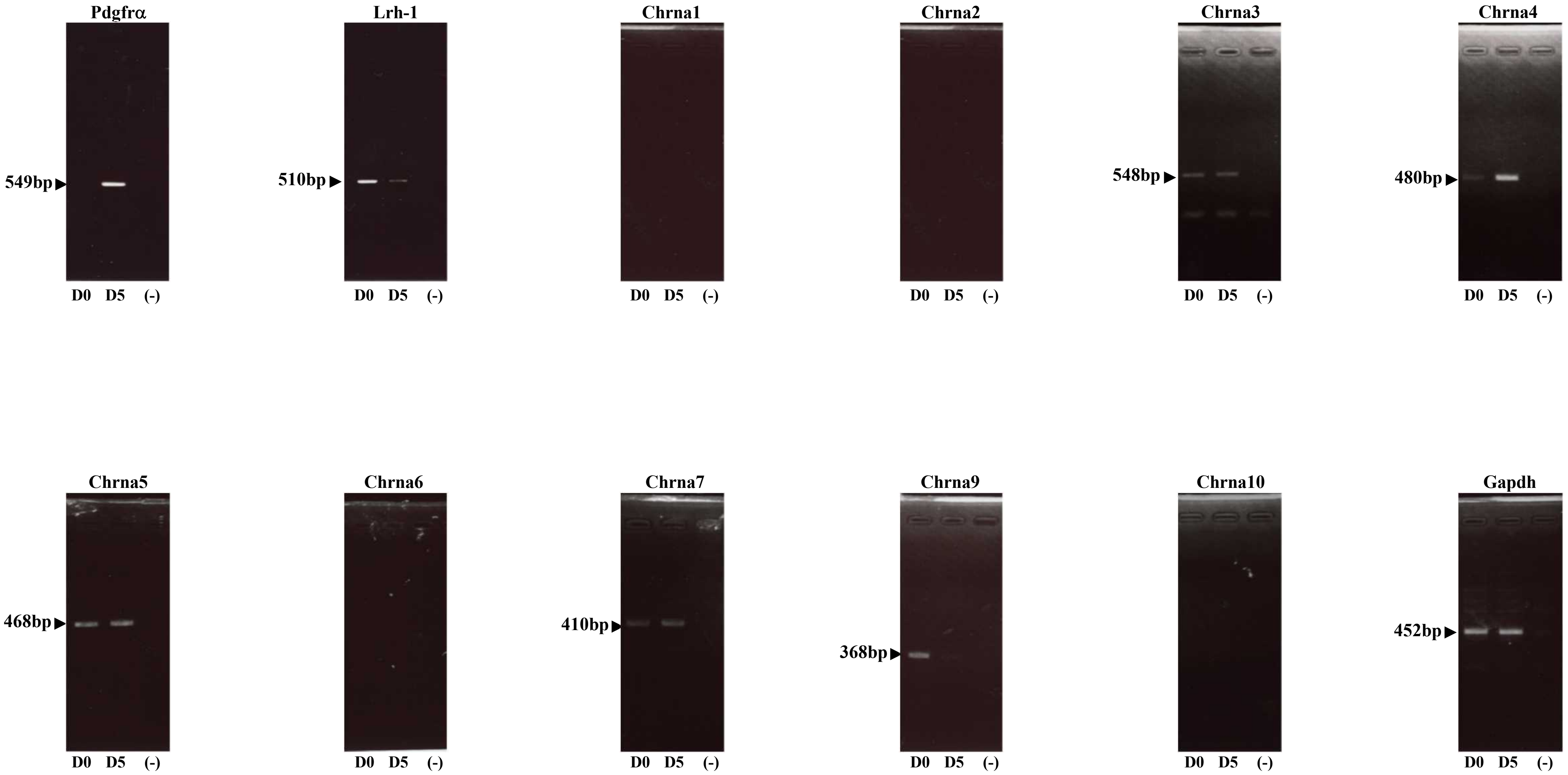

The whole images of RT-PCR analyses in Figure 1(A).

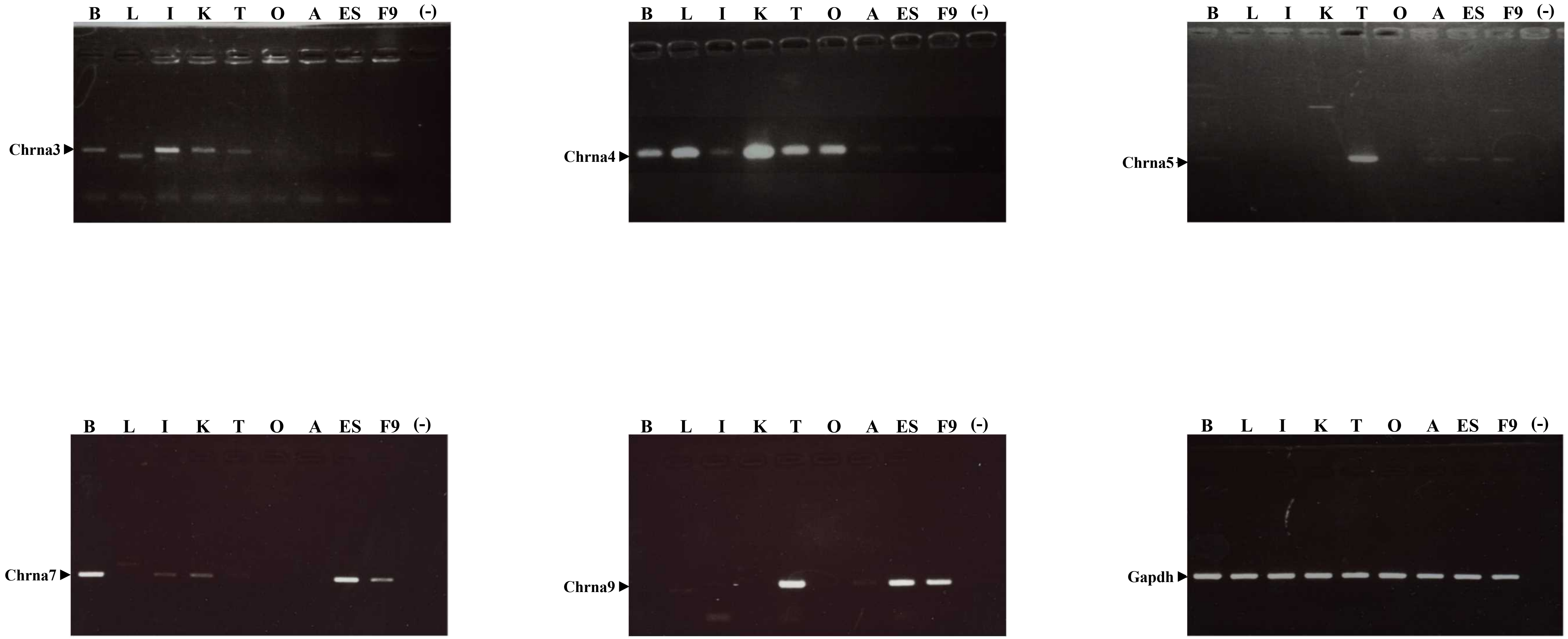

The whole images of RT-PCR analyses in Figure 2(A).

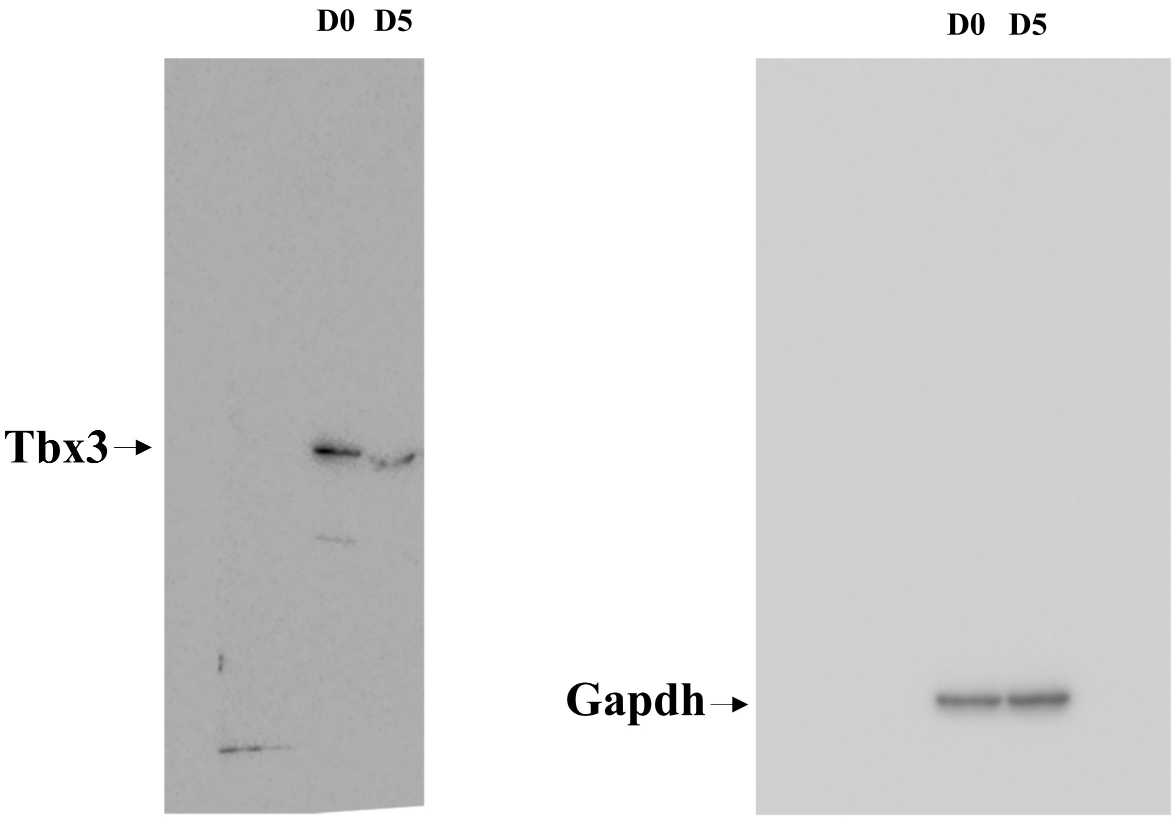

The whole images of western blot analyses in Figure 5(C).
